# How Did Social Medicine Evolve, and Where Is It Heading?

**DOI:** 10.1371/journal.pmed.0030399

**Published:** 2006-10-24

**Authors:** Dorothy Porter

## Abstract

A better understanding of how social medicine evolved, says Porter, could help to focus its role in responding to the health needs of a post-industrial, globalizing world.

## Introduction

The academic discipline of social medicine has struggled to find a precise definition for over a century. This struggle is exemplified by the classic social medicine course book, *The Social Medicine Reader*, edited by faculty from the Department of Social Medicine at the University of North Carolina, Chapel Hill, which offers an expansive view of social medicine's concerns [1–4]. These concerns range from early visions of the discipline, focusing on topics such as the social and economic structure of health-care provision, health policy, and clinical holism, through to evolving concepts of the field, such as concerns with doctor/patient relations in culturally diverse societies. The evolution of social medicine as an academic subject has been internationally diverse and a coherent definition of the discipline has remained elusive.

In this essay, I briefly examine some of the diverse developments of social medicine as an academic discipline and its links to political conceptualizations of the role of medicine in society. I then analyze the possible future directions open to the discipline in the Anglo-American context. A better understanding of the evolution of social medicine could help to focus its role in responding to the health needs of a post-industrial, globalizing world.

## The Interwar Years of the Twentieth Century: Programs of Social Reform

In 1945 René Sand was appointed as Professor of Social Medicine at Brussels University, one of the first professors appointed in this discipline. His post had been created by a financial endowment from the Rockefeller Fund. Sand believed that the roots of social medicine lay in ancient Greek philosophies of medicine and health [5].

Sand's younger contemporary, George Rosen, a historian and Professor of Public Health at Yale, traced the origin of the modern concept of the social role of medicine to the nineteenth century. Rosen emphasized the role of French and German health and social reformers, including Jules Guerin, Alfred Grotjahn, and, above all, Rudolph Virchow, the liberal politician and founder of cellular pathology [6,7].

Nineteenth-century health and social reformers had been concerned with developing the political role of medicine in creating egalitarian societies [8]. This concern continued to be a primary goal of twentieth-century medical academics, such as Sand, who wanted to integrate medicine's social role into the training of physicians through the creation of a new academic discipline of social medicine [9]. Virchow had articulated the need to develop a sociological method of inquiry into the conditions that maximized health and prevented disease [8]. Inspired by the experiments in sociological medicine and social hygiene in revolutionary Soviet society in the 1920s, interwar sociomedical reformers on both sides of the Atlantic believed that the creation of a sociopolitical role for medicine could be achieved by turning it into a social science [9].

The interwar years witnessed a wide variety of international developments in social medicine as an academic discipline. At Yale University, the Institute of Human Relations was created in 1931 under the direction of Milton Winternitz, the dean of the Medical School. The aim of the institute was to integrate medicine into research on social inequalities, which would inform the training of physicians to become, in Winternitz's words, “clinical sociologists” [10,11].

**Figure:**
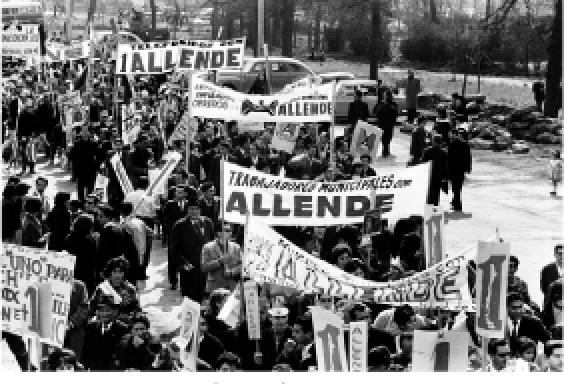
A march in support of Salvador Allende, who influenced the development of Latin American social medicine (Photo: US Library of Congress)

In the 20s and 30s Sand played a critical role in the international promotion of the new academic discipline of social medicine, especially in Latin America, where his work for the Rockefeller International Health Board supported the creation of social medicine institutes and departments at the University of San Marcos in Lima, Peru and the Oswaldo Cruz Institute of Rio de Janeiro in Brazil [12,13]. At the University of Chile, Max Westenhofer, a former Virchow student, taught social medicine as well as pathology to the future president of Chile, Salvador Allende. Allende developed a Marxist conceptualization which profoundly influenced the subsequent development of Latin American social medicine (LASM) and which was reflected in the creation of a national health service under his presidency in the 1970s [14].

Within international health organizations in the interwar years, supporters of social medicine as an academic discipline tried to undermine any exclusive focus on clinical medicine and pushed towards much broader social agendas. From the time of its establishment, the governing committee of the League of Nations Health Organization prioritized the development of social medicine. The International Labor Organization's representatives on the committee persistently argued that issues of social medicine could not be separated from the question of access to services that fundamentally affected the health of workers [15]. Before the Second World War, the International Health Committee of the Rockefeller Foundation also identified social insurance as a central issue of policy promotion.

As Paul Weindling has pointed out, the concern with developing multifactorial analyses of health and disease in the interwar period was stimulated by the economic crises of the 1930s and the effects of social deprivation [16]. Consequently many significant individuals in international health organizations, such as John A. Kingsbury and Edgar Sydenstricker from the Milbank Memorial Fund, saw social medicine as a question of health citizenship. This was also the case in national contexts. Within Britain, for example, the debates surrounding social medicine in the interwar years intersected with the debates surrounding the planning of a national health service and the establishment of free access to services at the point of delivery as a fundamental social right of democratic citizenship [16].

**Figure:**
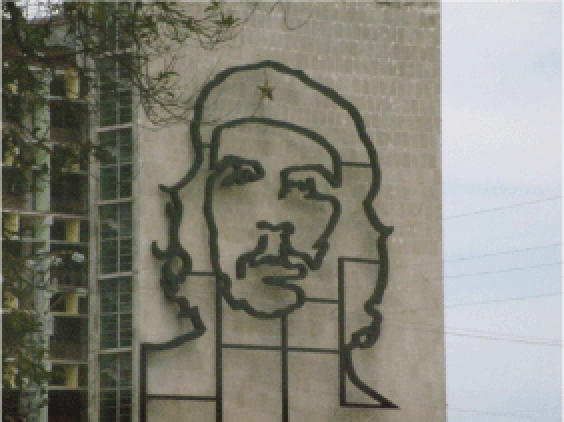
Che Guevara's “revolutionary medicine” stressed the social origins of illness and the need for social change to improve health conditions (Photo: Marko Faas)

The goals of social medicine as an academic discipline as it developed in the interwar years, therefore, were overtly linked to political programs of social reform. The international social medicine movement before the Second World War aimed to create a new social role for medicine in order to grapple with the epidemiological transition created by economic and social developments in the twentieth century. The interdisciplinary program between medicine and social science would provide medicine with the intellectual skills needed to analyze the social causes of health and illness in the same way as the alliance between medicine and the laboratory sciences had provided new insights into the chemical and physical bases of disease. But these developments took place within, and were inherently bound to, the international debate concerning the establishment of socialized medicine and the eradication of health and socioeconomic inequalities.

## From Social Structure to Social Behavior: The Rise of Lifestyle Medicine

The institutionalization of social medicine after the Second World War varied widely among different national contexts. Two contrasting examples are LASM and social medicine in the Anglo-American environments.

### The Latin American context: A focus on the social and structural determinants of health.

Following the Second World War, LASM became increasingly differentiated from public health as an academic discipline by virtue of its aim to embed Marxist and post-Marxist social and political theory into research and teaching. Latin American social medicine focused on political and social transformation, whereas public health continued to prioritize the practical implementation of public policy as a central empirical and intellectual goal.

An example of the way in which LASM focused on social transformation is Ernesto (“Che”) Guevara's conceptualization of “revolutionary medicine,” which hinged on the training of all health-care professionals, including physicians, in the social origins of illness and the need for social change to improve health conditions. Che Guevara's reflections played a profound role in the Cuban, Chilean, and Nicaraguan revolutionary governments' reform of medical and health-care systems and education [14].

These developments were further extended by an emergent leader of LASM in the 1970s, Juan César García, who had trained as a physician in Argentina and as a sociologist in Chile. García was a research coordinator within the Pan American Health Organization from 1966. In the late 70s and early 80s he organized a series of social medicine seminars, raised and distributed grants, contracts, and fellowships throughout the region, and published numerous volumes on social medicine [17]. In 1984, the year he died, leading social medical reformers from within his seminar group created the Latin American Social Medicine Association (ALAMES) which became an academic and political presence in all countries throughout the continent.

ALAMES promoted a cohesive conceptual foundation for social medicine in medical education in each of these hugely varying national contexts grounded in contemporary socialist theories of health and society [18,19]. Thus, social medicine in Latin America continued to directly engage with major theoretical and methodological debates within the social sciences and within Marxism that explored the social structural determinants of disease such as economic inequality.

### The Anglo-American context: The rise of “lifestyle medicine.”

In contrast to LASM, social medicine in the Anglo-American context developed a model of prevention that primarily focused on changing *individual* behavior rather than addressing the social structural determinants of health and disease. Unlike LASM, Anglo-American social medicine struggled to become institutionalized as an academic discipline which influenced medical education.

In the United States, social medicine was a casualty of the ever widening gap between preventive and therapeutic medicine. Alan Brandt and Martha Gardner have pointed out the reasons for the widening and often increasingly hostile divide between medicine and public health in the US from the beginning of the twentieth century. These reasons included contrasting theoretical perspectives on disease control and management, conflicting goals of professionalization, and the rise of medical authority with the expansion of hospital-based specialist practices. [20].

Brandt and Gardner argue that following the Second World War a new accommodation was achieved, however, within US public health as an academic discipline and as a professional practice, as public health adopted a more biomedical rather than sociomedical model of disease within a preventive philosophy driven by the management of individual risk factors for chronic illnesses [20]. Their argument is powerfully illustrated in the analytical frameworks that drove preventive strategies for cardiovascular disease, lung cancer, and obesity. These frameworks were supported by the development of what Gerald Oppenheimer has argued was largely a behaviorist model of clinical epidemiology in the early post-war decades [21,22].

From the 1920s, statisticians working in the US life insurance industry had begun to examine the relationships between lifestyle, overweight, and cardiovascular morbidity and mortality [23–27]. At the end of the war the US Public Health Service initiated new studies of the impact of the epidemiological transition to chronic diseases when Joseph Mountain hired Gilcin Meadors in 1946 to found what eventually became the Framingham study of heart disease in 1947 [21]. Meadors set up the initial study with the expressed purpose of producing “recommendations for the modification of personal habits and environment” that could prevent the development of coronary heart disease [21].

While the Framingham study highlighted the role of diet and cholesterol, by the early 1950s in Britain Jerry Morris and his colleagues at the Medical Research Council Social Medicine Unit were highlighting another lifestyle determinant of coronary heart disease: exercise [28]. In the meantime, in 1948 Iwao Milton Moriyama and Theodore Woolsey produced a large analysis of cardiovascular disease in relation to age changes in the population using population survey data that also included discussions of lifestyle issues such as obesity [29].

In October 1952, the National Vitamin Foundation funded a symposium at Harvard University on overeating, overweight, and obesity which included papers on lipogenesis, the psychology of overeating, the physiology of overweight, and a paper by P.C. Fry on “Obesity: Red Light of Health” [30]. The public and individual health implications of overweight and obesity attracted increasing attention throughout the 1950s. Numerous public health authors took up the issue of overweight with such titles as *Your Weight and Your Life* and *The Low-Fat Way to Health and Longer Life: The Complete Guide to Better Health through Automatic Weight Control, Modern Nutritional Supplements, and Low-Fat Diet* [31,32]. Psychology research students undertook studies such as “Dimensions of Personality as Related to Obesity in Women” [33].

In Britain, such behavioral analysis also began to replace traditional structural (political/economic) explanations of core public health concerns such as infant mortality. Since the nineteenth century, studies of infant mortality had prioritized economic inequality as the major cause of steep differential gradients according to class. However, new sociobehavioral investigations began to explore other factors in the early 1950s. Because at that time it was extremely difficult to determine the intrauterine events that may have led to the death of babies within the first four weeks of life, often the cause of death on certificates was simply listed as “prematurity.”

**Figure:**
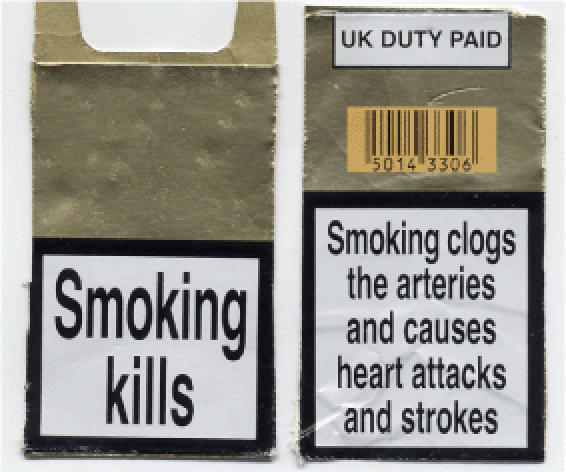
Doll and Hill's research led to an anti-smoking campaign in Britain that exemplified a behavioral model of chronic disease prevention (Photo: Adrian Pingstone)

Stewart, Webb, and Hewitt from the Oxford Institute of Social Medicine suggested that this term really described a way of dying rather than an actual cause. In 1955 they attempted to correlate 1,078 stillbirths and neonatal deaths with a variety of factors, including the mother's physique during the antenatal period [34]. The result of investigating what would appear to be the biological conditions pertaining to death resulted, however, in identifying social behavior as a major factor. In their 1955 study Stuart, Webb and Hewitt discovered that:

*“Medium” and “thin” women did not differ in their ability to produce live infants, but among the 212 women described as “obese” the risk of still birth or neonatal death was 60 per cent above the standard. This risk appeared to be still greater among the women who were described as both “obese” and “short” [34].*

The established structural explanation of the relationship between poverty and infant mortality was thus challenged by a new behavioral argument about mothers' obesity as the major determinant of stillbirths and neonatal deaths. This new argument claimed that *lifestyles*, involving unhealthy behaviors such as excessive food consumption and lack of exercise, created major risks rather than *life conditions* such as economic inequality.

One of the most dramatic early correlations of behavioral lifestyle habits and chronic illness in Britain was established by Richard Doll and Austin Bradford Hill in their analysis of cigarette consumption and rising levels of lung cancer published in the *BMJ* in 1950 [35,36]. Both Doll and Hill were founding members of the British Social Medicine Association when it was set up in 1957 and were closely involved with the social medicine intellectual community. Although smoking was considered a habit, rather than a dependency in the strict psychological definition of addiction, it was represented as an individual responsibility [37,38]. The anti-smoking campaign in Britain which followed the Doll and Hill results exemplified the new message of a behavioral model of chronic disease prevention. The key to the social management of chronic illnesses—such as lung cancer—was through changing individual behavior, raising health consciousness, and promoting self–health care.

Following the anti-smoking campaign, the strategy of preventing chronic disease through education of individual behavior gathered momentum in the Anglo-American context. Subsequent post-war campaigns offered behavioral, lifestyle methods for preventing heart disease, various forms of cancer, liver disease, digestive disorders, venereal disease, and obesity. The behavioral model of lifestyle prevention became indistinguishable from social medicine as an academic discipline throughout this period as it was increasingly grounded in a methodologically individualist epidemiology as the dominant science of etiology of chronic disease.

**Figure:**
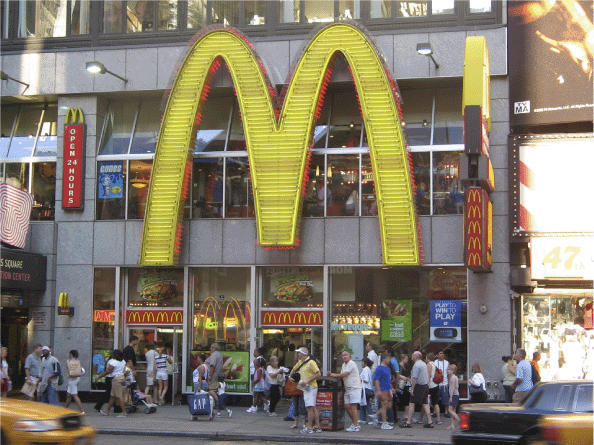
Kelly Brownell has suggested that the marketing and advertising techniques of the corporate food industry play a role in the rise in obesity

The analysis of the relationship between smoking and lung cancer gave epidemiology a new legitimacy as the scientific key to revealing a bio-psycho-socio-medical model of chronic illness. The latter became a critical explanatory model underwriting a new approach to disease prevention through the control of individual lifestyles. The methodologically individualist basis of social medicine, with its focus on prevention of disease through lifestyle and behavior changes, mirrored the individualist focus of therapeutic medicine. Together, preventive and therapeutic medicine expanded a behavioral and individualist model of the relationship between health and disease in the latter half of the twentieth century [39].

## A New Direction for Anglo-American Social Medicine: A Return to a Focus on Social Origins of Disease?

Social medicine in the Anglo-American context remained closely bound to the bio-psycho-socio-model of chronic disease that underpinned lifestyle-based, behavioral models of prevention after the Second World War. However, recent political developments in chronic disease prevention, specifically with respect to tackling obesity, may encourage yet another paradigmatic shift. This shift may re-link Anglo-American social medicine with its roots in social structural analysis of disease etiology and with the continued socioeconomic structural analytical focus of LASM.

As discussed above, since the Second World War obesity figured prominently in the development of a behavioral etiological framework for analyzing chronic diseases and in a behavioral conceptualization of prevention through individual lifestyle changes. Since the 1990s, however, this conceptual framework has been challenged by politically and intellectually radical health reformers who have focused on the role of corporate capitalism in the production of diseases. Radical health discourses since the 1990s have focused on deconstructing the neo-liberal model of prevention as an individual responsibility. For a much longer period, the role of corporate industrial organizations in the production of chronic disease has been highlighted by historians of occupational diseases [40,41] and by historians of smoking together with reformers' and government agencies' battles with the tobacco industry from the 1980s [42,43]. More recently, a radical health reform war on “Big Food” has mimicked many of the strategic arguments and actions of the earlier wars on “Big Tobacco” and “Big Pharma.”

In their war against “Big Food,” radical health reformers have rejected the behavioral etiological explanation of obesity and have focused instead on an environmental explanation. Ironically, one of the foremost supporters of this idea has been a distinguished behavioral scientist, Kelly Brownell, who has suggested that the cause of obesity and overweight is a toxic environment of addictive food production, which includes the mass marketing and advertising techniques of the corporate food industry [44]. Brownell and his co-founders of a health reform group, Center for Science in the Public Interest, have argued for government intervention to control and prevent obesity through taxation of high-calorie foods of low nutritional value, the banning of junk food sales from schools, and compulsory public disclosure of calorific and chemical components of nutritional products, including on restaurant menus [45, 46].

The environmental analytical framework offered by the reform “food warriors” has been endorsed by the most recent investigations of the World Health Organization in their 2003 *Draft Global Strategy on Diet, Physical Activity and Health* [47]. The draft strategy recommends that the governments of the most affluent societies introduce nutritional taxation to supplement the production and sales of nutritious foods, impose legal controls on the sale of non-nutritious high-calorie products to children, and regulate the marketing and advertising activities of the food industry to protect vulnerable populations. A special emphasis is made in the draft strategy on the need to prevent the corporate food industry from attempting to get children addicted to certain foods as a marketing mechanism (a mechanism that was used by the tobacco industry to addict children to cigarettes).

## A New Framework of Disease Etiology for Social Medicine

Social medicine researchers and academic educators face the task of integrating the historical wisdom acquired during the evolution of the discipline into a new framework. This framework is needed for understanding the complex interaction of biology with the political, economic, social, and cultural relations of the twenty-first century.

The most recently expanded environmental and social structural etiological models of chronic illness revealed in the course of the “obesity wars” offer a possible route to a new shift for social medicine. These recent models reconnect the discipline with the socioeconomic structural analytical frameworks on which it was originally founded and also further redefine them in the context of the corporate structure of economic production in the twenty-first century. At the same time, such an intellectual shift would integrate the future evolution of social medicine in the Anglo-American with the LASM holistic socioeconomic structural model of disease etiology. A greater integrated international social medicine discourse may be the best fit to confront the challenges of understanding a new global order of health, disease, medicine, and the disparities of care and resources.

## Abbreviations

LASM: Latin American social medicine
